# Biomarker-Guided Drug Delivery Systems and Oral Bioavailability Enhancement

**DOI:** 10.3390/ph19030454

**Published:** 2026-03-11

**Authors:** Dang-Khoa Vo, Van-An Duong

**Affiliations:** 1 College of Pharmacy, Gachon University, 191 Hambakmoe-ro, Yeonsu-gu, Incheon 21936, Republic of Korea; 2 The Brown Foundation Institute of Molecular Medicine, McGovern Medical School, The University of Texas Health Science Center at Houston, Houston, TX 77030, USA; van.an.duong@uth.tmc.edu

**Keywords:** biomarker-guided therapy, oral bioavailability, targeted drug delivery, nanomedicine, personalized medicine, drug absorption enhancement

## Abstract

Biomarker-based guided delivery of drugs is an emerging paradigm of precision medicine in which targeted therapeutic intervention is administered on the basis of certain biological markers in order to achieve maximal dosing, targeting, and time optimization. By utilizing quantifiable physiological or molecular signatures like the expression of transporters, enzymatic activities, metabolite levels, or disease-specific markers to tie in the correlation of drug disposition, these systems provide individualized intervention with optimized efficacy and safety. Oral administration of drugs is still the best route in patient compliance; however, several drugs are handicapped by suboptimal bioavailability secondary to poor solubility, limited permeability, efflux transporter participation, and enzymatic first-pass degradation. These result in variable therapeutic results in patient populations. Biomarker guidance in oral drug delivery provides a potent strategy for overcoming such challenges through site-specific release, real-time dose optimization, and adjustment of absorption pathways. Recent developments include pH-controlled formulations for gut-specific targeting, enzyme-activated nanocarriers, glucose-starved responsive devices for metabolic disease, and biomarker-driven transporters for permeability enhancement. Preclinical and early-phase clinical studies hold promising prospects for applications in oncology, infectious disease, inflammatory bowel disease, and metabolic disease. While promising momentum exists, transition to routine use in the clinic awaits rigorous biomarker validation, scalability in manufacture, and regulations harmonization. On the horizon, the integration of biomarker-guided oral drug delivery with nanotechnology, artificial intelligence, machine learning, and wearable biosensors holds promise for revolutionizing oral therapy into very personalized, responsive, and efficient treatment methods.

## 1. Introduction

Over the past two decades, drug delivery platforms have transitioned from traditional one-size-fits-all formulations to precision medicine approaches, corresponding to the distinctive requirements of individual patients [1]. Existing formulations are specially engineered for stability, convenience in dosing, and prolonged circulation lifetimes, but they do not consider interpatient pharmacokinetic, pharmacodynamic, and therapeutic response variability [2,3]. The emergence of precision medicine has driven the design of delivery platforms capable of targeting specific tissues, releasing drugs in response to physiological cues, and adapting to dynamic disease states [4]. At the very center of such transformation remains the increased utility of biomarkers that are employed for stratifying patients on the basis of the benefit corresponding to a particular therapeutic agent [5]. Biomarkers are not merely employed for the identification of responsive subpopulations but for real-time measurement of therapeutic and toxic effects in vivo [6]. Through the implementation of biomarker profiles in the engineering of the delivery system, scientists are able to custom-tune the release kinetics of drugs, site of targeting, and dosing in correspondence with the patient’s distinctive biological environment [7,8]. Such evolution remains a defining stride towards optimization of therapeutic effect, minimization of undesirable effects, and maximization of overall clinical effect, ultimately filling the gap in transition from molecular diagnostics towards customized drug delivery.

Oral administration continues to provide the most convenient and most common route for drug administration owing to its non-invasive nature, simple administration from a self-care standpoint, and high patient compliance [9]. Optimal therapeutic effects via oral administration are, however, too often frustrated by various biopharmaceutical and physiologically determined absorption limitations [10]. A key limitation is low aqueous solubility, which limits dissolution and decreases the concentration of the drug available for absorption [11,12]. Low permeability through the intestinal epithelial layer further imposes limits on system exposure for hydrophilic or large entity therapeutics [13,14]. The other drugs are also prone to enzymatic or chemical instability in the gastrointestinal (GI) environment due to acidic gastric pH, digestive enzymes, and microbial metabolism-mediated pre-systemic degradation [15]. Furthermore, extensive intestinal mucosa and liver first-pass metabolism remove most of the active drug from systemic circulation [16]. Interpatient variability in absorption, genetic polymorphism, regulation of transporter expression, gut microbiota population, and disease-modulated regulation of GI physiology make dose optimization challenging [17,18]. These challenges are generally responsible for low and inconsistent bioavailability, requiring increased doses that enhance the risk of side effects [19]. Such difficult challenges have to be resolved by new ideas in formulation and targeted delivery systems that can enhance solubility, increase permeability, shield drugs from inactivation, and control metabolism to achieve stable therapeutic effects [20].

Integration of biomarkers into oral drug delivery design presents a revolutionary route to optimizing therapeutic performance using the promise of personalized medicine [21]. Biomarkers, from genetic polymorphism and protein expression profiles to metabolic fingerprints and physiological metrics, have the potential to give useful information regarding interindividual variability in drug absorption, distribution, metabolism, and elimination [22]. By correlating certain biomarkers with pharmacokinetic events, including transporter activity or enzyme-catalyzed metabolism, formulation scientists can better predict and optimize drug exposure in every individual patient [23]. Likewise, pharmacodynamic biomarkers, mirroring effects of the drug on biology, can inform dose titration and therapeutic monitoring such that drug levels are kept within the optimal efficacy-safety zone [24]. With this intersection of oral therapy, the possibility is created to design delivery systems that dynamically respond to alterations in biomarkers to release the drug at the correct site, time, and rate to coincide with the patient’s own individual biological profile. For instance, permeability enhancement strategies can be guided by biomarkers of transporter expression [25], and metabolic biomarkers can direct enzyme-inhibiting co-formulations to avoid first-pass metabolism [26]. This biomarker-referenced precision not only maximizes bioavailability but also minimizes variability of the therapeutic response, minimizes side effects, and maximizes the outcome of treatment, closing the gap between diagnosis and customized oral drug delivery [27].

A blend of biomarker-directed approaches with oral bioavailability optimization is a new paradigm that is rapidly developing at the convergence of personalized medicine, novel formulation science, and therapy optimization [28]. Although the oral route is best suited for the majority of patients, traditional methodologies neglect the extreme heterogeneity between individuals in drug disposition due to genetic, molecular, and physiological causes [29]. Biomarker-guided technologies can revolutionize this landscape by allowing responsive, targeted, patient-specific oral delivery, which may respond and adapt drug release and absorption to the own biologic fingerprint of the individual patient [30]. This review provides an integrated overview of how biomarkers in pharmacokinetic, pharmacodynamic, predictive, and safety markers may be applied to tackle critical oral delivery issues such as poor solubility, low permeability, instability, and high first-pass metabolism. We first explain the basic principles of biomarker detection and classification, and discuss oral drug delivery issues and the mechanistic function of biomarkers as their solutions. We then summarize existing biomarker-responsive release technologies, formulation-integrated approaches for enhancing oral bioavailability, and case studies for documenting in vivo relevance. This review also includes a discussion on regulations, challenges in translation, and prospects, where the promise of a synergistic combination of biomarker-driven formulation and next-generation oral drug release technologies is highlighted. The objective of this review is to develop a mechanistic and translational basis to connect biomarkers to oral drug delivery design. We describe the major physiological, biochemical, and disease-related barriers to drug absorption, metabolism, and excretion in the oral delivery context. We then evaluate the role of validated endogenous or pathological biomarkers, including transporters, metabolic enzymes, inflammatory markers, and microbiome markers, in quantitatively measuring these barriers. Rather than focusing on a wide range of delivery vehicles, we will highlight the opportunities for rational design of drug delivery vehicles, including stimulus-responsive, targeted, and adaptive delivery vehicles, in the context of what can be learned from biomarkers. Unlike previous reviews that address stimuli-responsive systems or general oral drug delivery barriers individually, this review uniquely brings together endogenous and disease-related biomarkers with rational formulation design to create a new paradigm for the translation of biomarker-guided oral drug delivery systems. We specifically discuss clinically relevant transporter and enzyme biomarkers (e.g., taurine, pyridoxic acid, creatinine), inflammatory mediators, and microbiome signatures as mechanistic tools for addressing absorption and metabolism-related barriers, predicting drug–drug interactions, and addressing interindividual variability. This review uniquely brings together the science of biomarkers with formulation science to create a new paradigm for the translation of precision medicine for the next generation of oral therapeutics.

## 2. Literature Search Strategy

The literature search strategy employed for this review article is a structured narrative search strategy. The literature was searched by entering relevant keywords related to the topic in the PubMed, Web of Science, and Scopus databases up to February 2026. The keywords used for the literature search are related to “oral drug delivery”, “biomarkers”, “endogenous biomarkers”, “drug transporters”, “CYP450”, “UGT”, “OATP”, “OCT”, “OAT”, “stimuli-responsive systems”, “drug–drug interaction”, “biomarker-guided therapy”, “oral bioavailability”, “targeted drug delivery”, “nanomedicine”, “personalized medicine”, “drug absorption enhancement”, and “precision medicine”. Emphasis was placed on peer-reviewed literature related to original research articles, clinical studies, and high-impact review articles that are mechanistically relevant to the topic. The literature search strategy followed contemporary scientific standards by focusing on literature published from 2015 onwards, except for foundational literature that remains highly relevant to the topic.

## 3. Fundamentals of Biomarkers in Drug Delivery

### 3.1. Classification of Biomarkers Relevant to Drug Delivery

**Figure 1:**
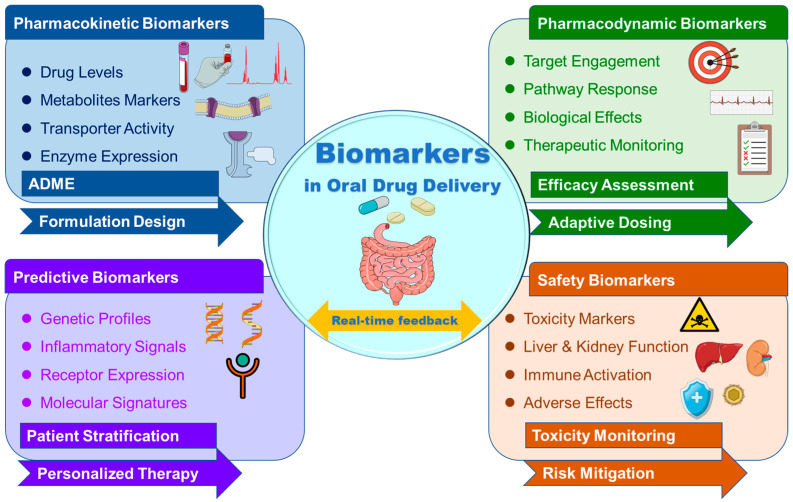
Integrated classification and functional roles of biomarkers in biomarker-guided oral drug delivery. Created using Mindthegraph.com and Microsoft PowerPoint 2019.

Biomarkers, as strictly objective indicators of normal or pathological biological processes or pharmacological responses, are unmatched assistants in guiding drug development, therapeutic monitoring, and personalized delivery plans [31]. In the context of drug delivery, biomarkers are categorized into pharmacokinetic, pharmacodynamic, predictive, and safety biomarkers, each providing distinct insights into drug disposition and therapeutic endpoints [27]. Pharmacokinetic biomarkers, such as the level of expression of transporters or metabolite profiles, offer inputs for drug absorption, distribution, metabolism, and excretion parameters, allowing a prediction of system exposure [32]. Pharmacodynamic biomarkers are a measure of the biological effect of a drug, allowing real-time therapeutic optimization [33]. Predictive biomarkers are those that determine patients likely to benefit from a given therapeutic intervention [34], while safety biomarkers are early predictors of toxicity or undesirable effects [35]. Application of biomarkers in drug delivery goes beyond patient selection to system engineering, capable of responding dynamically to certain physiological or molecular stimuli, such as pH change, enzyme activity, or metabolite levels, thus allowing site-specific and controlled release [36,37]. Integration of biomarker science with technology in delivery offers a basis for precision therapeutics, especially in oral administration, in which interpatient variability readily nullifies uniform efficacy [38]. Comprehension of biomarker classification, relevance, and detection holds the key to designing the next generation of drug delivery platforms. Figure 1 shows the schematic illustration of key biomarker classes relevant to biomarker-guided oral drug delivery systems. Pharmacokinetic biomarkers, such as the concentration of drugs or their metabolites, and transporter activity markers are used to monitor the process of absorption, distribution, metabolism, and excretion. Pharmacodynamic biomarkers, which reflect the biological response, are used to monitor the pharmacodynamics of the drugs. Predictive biomarkers are used to stratify patients and predict the response to the drugs, and safety biomarkers are used to monitor the safety of the drugs.

### 3.2. Analytical and Detection Methods

Accurate detection and quantification of biomarkers are essential for their fruitful application in designing drug delivery systems [39]. Advanced analytical technologies enable the detection of molecular, cellular, and physiologic markers with high sensitivity, specificity, and temporal resolution [40]. Mass spectrometry-based instruments, such as liquid chromatography–tandem mass spectrometry (LC-MS/MS), remain the gold standard for quantifying pharmacokinetic and metabolic biomarkers due to their high sensitivity and specificity in detecting low-abundance entities in complex biological mixtures [23,41]. Immunoassays, including enzyme-linked immunosorbent assays (ELISAs) and multiplex bead-based assays, are the preferred methods for detecting proteins and cytokines in pharmacodynamic monitoring [42]. Genomic and transcriptomics tools, like next-generation sequencing (NGS) and quantitative polymerase chain reaction (PCR), are valuable for profiling the expression of drug transporters and metabolizing enzymes, as well as identifying genetic polymorphisms [43,44]. Innovative biosensor technologies, particularly implantable and wearable sensors, facilitate real-time monitoring of dynamic biomarkers such as glucose, lactate, or inflammatory mediators in response to drug release [45]. Imaging technologies like positron emission tomography (PET) and magnetic resonance imaging (MRI) can track disease-related molecular signatures and drug distribution in vivo [46]. When these analysis platforms are integrated into drug release systems guided by biomarkers, they enable precise patient stratification, real-time therapy optimization, and critical evaluation of formulation performance in both preclinical and clinical settings.

### 3.3. Biomarkers Influencing Oral Drug Disposition

Oral disposition is regulated primarily by physiological and molecular biomarkers controlling absorption, metabolism, and elimination [47]. Heterogeneity of transporter expression due to genetic polymorphisms, disease states, or drug–drug interactions can be exploited as a predictive biomarker for oral exposure and sensitivity [48]. For instance, Sahoo et al. [49] determined the impact of transporter gene polymorphisms on treatment outcomes and toxicity in a total of 120 oral cancer patients who received Taxane/Platinum/5-fluorouracil (TPF) or Paclitaxel/Carboplatin (PC) chemotherapy. The study found that ABCG2 rs4693924, ABCC2 rs2804398, ABCC4 rs943288, and ABCC1 rs9332430 polymorphisms are associated with certain adverse events of chemotherapy, including anemia, diarrhea, dysphasia, and nausea. No significant relationship was found between the polymorphisms and progression-free survival. The study demonstrated that transporter gene polymorphisms are associated with drug disposition-related toxicity rather than drug efficacy. This suggests that they can be used as predictive markers for the management of oral chemotherapy.

Enzyme activity biomarkers, notably those correlating to cytochrome P450 isoforms (e.g., CYP3A4, CYP2C9) and phase II enzymes, including UDP-glucuronosyltransferases (UGTs), affect the extent of first-pass metabolism and the systemic drug concentration [50]. Hashiba et al. [50] explored the expression, activity, and inducibility of cytochrome P450 (CYP) and UDP-glucuronosyltransferase (UGT) enzymes in 3D-cultured human renal proximal tubule epithelial cells (3D-RPTEC) as an in vitro model of renal drug metabolism. In comparison with 2D cultures, 3D-RPTEC displayed significantly increased expression of CYP2B6, CYP2E1, CYP3A4/5, and UGT isoforms, which is similar to that found in human kidney cortex. Increased metabolic activity and inducibility by nuclear receptor ligands were also observed. This study shows that the kidney possesses considerable metabolic potential and that differences in CYP/UGT expression and induction in the kidney may be crucial biomarkers for oral clearance of drugs, drug interactions, and pharmacokinetic differences among individuals. Physiological biomarkers such as gastrointestinal pH, bile salt concentration, gastric emptying rate, and gut microbiota composition also affect dissolution, solubility, and metabolic stability [51]. Under certain pathophysiological conditions, disease-specific biomarkers such as inflammatory cytokines in inflammatory bowel disease or diabetic glucoses in diabetes may offer a possibility of being harnessed as triggers for site-specific or condition-dependent release of drugs [52]. A recent study by Jiang et al. [53] has revealed that NEDD4-binding protein 3 (N4BP3) is a critical regulator of intestinal inflammation through the activation of the TLR4-NF-κB signaling pathway by increasing the ubiquitination and degradation of IκBα. This knowledge of the inflammatory signaling pathways points to the role of inflammatory signaling cascades as functional biomarkers of disease progression in IBD, thereby emphasizing the role of inflammation-responsive oral drug delivery systems that use pathway-level biomarkers such as inflammatory signaling cascades as triggers for action. Additionally, a comprehensive review of recent transcriptomic analyses by Li et al. [54] has emphasized the role of AP-1/FOSL1-associated transcriptional regulation in modulating inflammatory signaling and cellular plasticity. These upstream regulatory networks influence cytokine expression, epithelial integrity, and stress responses, factors directly impacting drug disposition and therapeutic efficacy. Incorporating transcriptional network biomarkers into delivery system design broadens the biomarker framework beyond circulating mediators, enabling pathway-informed stratification and more precise prediction of therapeutic responsiveness. Elucidation of such biomarkers facilitates the rational design of oral delivery platforms to bypass, modulate, or sense these determinants and, in turn, eliminate interpatient variability and achieve maximized oral exposure in a predictable and individualized manner.

## 4. Overview of Oral Bioavailability Challenges

### 4.1. Physiological Barriers, Pathophysiological, and Interpatient Variability

Oral bioavailability is the percentage of an oral dose entering systemic circulation in an active state. It is generally impaired by a series of physicochemical, physiological, and biochemical obstacles [55]. Low aqueous solubility, as for most Bio-pharmaceutics Classification System (BCS) Class II and IV compounds, limits dissolution in GI fluids, thereby restricting absorption [56]. Although solubility is optimal, low membrane permeability may block transcellular or paracellular transport across the intestinal epithelium [57]. Chemical instability in the acidic gastric or enzymatic hydrolysis by luminal and brush-border enzymes further decreases the fraction of intact drug remaining for absorption [58]. After uptake, drugs become a victim of extensive first-pass metabolism by intestinal tissue and liver through metabolic enzymes like cytochrome P450s and conjugating enzymes, leading to extensive loss of active compound [59]. Efflux transporters like P-glycoprotein also actively pump the drugs back into the intestinal lumen, again reducing net absorption [60]. Due to genetic polymorphisms, diet, composition of the gut microbiota, age, and disease state, interpatient variability presents an additional factor in uncertainty for oral drug exposure [61]. Multifactorial barriers present a stimulus for sophisticated formulation approaches, and with the assistance of biomarker-guided design, a pathway to bypassing variability, improved absorption, and therapeutic outcome. A study by Akhilesh et al. [62] developed a cationic liposome nanoformulation targeting the TRPA1 receptor with siRNA to treat chemotherapy-induced peripheral neuropathy (CINP). CINP is a complex disease process that results in the upregulation of the TRPA1 receptor and the resultant neuroinflammation. The encapsulation of siRNA into a liposomal nanoformulation enables it to overcome the natural barriers that would otherwise limit its effectiveness as a therapeutic agent. The study showed that the IV and its routes were effective in the treatment of CINP, as the IV route exhibited the greatest silencing effect and anti-inflammatory properties. The modulation of the TRPA1 receptor and the resultant inflammatory markers, such as IL-6 and ICAM-1, allows the nanoformulation to overcome the pathophysiological variations that occur in the complex disease process.

### 4.2. Role of Biomarkers in Identifying These Barriers

Biomarkers have a crucial role to play in untangling and solving the intricacies of oral drug delivery. Through offering measurable indicators of transporter expression, metabolic enzyme activity, gastrointestinal function, and disease status, biomarkers enable enhanced understanding of the mechanistic causes of drug absorption and metabolism variation. A representative case study on the application of the approach to overcome transporter-related barriers using the approach of identifying and characterizing the effects of a compound on transporters using endogenous biomarkers as a model system has been demonstrated in our recent study on the effects of 1α,25-dihydroxyvitamin D_3_ (calcitriol) on the modulation of organic anion transporters Oat1 and Oat1/3 [63]. In the study, the effects of the compound on the plasma concentration, excretion, and tissue distribution patterns were demonstrated using the endogenous biomarkers taurine and pyridoxic acid, which were sensitive to the effects on OAT1/3 transporters as observed in the comparison with the effects on the pharmacokinetics of methotrexate. Our findings support biomarker-based DDI assessment for clinical translation. Complementary evidence to support the usefulness of endogenous transporters as biomarkers in the assessment of disposition-related issues is provided in two related studies in which calcitriol caused significant downregulation of the expression of renal OCTN1, OCTN2, and OCT2 transporters, which in turn caused marked changes in the pharmacokinetics and biodistribution of ergothioneine, L-carnitine, and creatinine [64,65]. These changes were characterized by increased plasma exposure, reduced renal clearance, and reduced tissue distribution, which are indicative of the inhibition of transporters. The high correlation between the suppression of mRNA and the observed pharmacokinetic and biodistributional changes further supports the notion that the endogenous compounds, namely ergothioneine, L-carnitine, and creatinine, are sensitive biomarkers of OCTN1, OCTN2, and OCT2 transporters, respectively. In addition, efflux transporter biomarkers like P-glycoprotein and organic anion-transporting polypeptide (OATP) levels predict efflux-mediated poor bioavailability in sensitive patients and direct the administration of permeability enhancers or transport inhibitor use [66]. For example, the utility of endogenous biomarkers in the identification of transporter-mediated barriers was further exemplified by coproporphyrin I (CP-I) as a sensitive biomarker of OATP1B1 inhibition. In a clinical study of glecaprevir/pibrentasvir [67], the CP-I plasma exposure, as measured by Cmax and AUC, was found to be proportional to the inhibitor concentration, showing a strong correlation with the extent of OATP1B1 inhibition, which was better than CP-III. Most importantly, the CP-I exposure was found to be correlated with the static DDI prediction parameters, which would be useful in the meaningful assessment of transporter inhibition without the need to conduct DDI studies. Likewise, enzymatic biomarkers for cytochrome P450 or UGT activity determine avoidance tactics for first-pass metabolism, e.g., co-administration with enzyme inhibitors or prodrug design [68,69]. Further evidence of the role of endogenous biomarkers in assessing metabolic barriers was sought by investigating 4βHC and 6βHCL as endogenous biomarkers of constitutive CYP3A activity. In a study of healthy subjects given an oral microdose of midazolam, significant variability of CYP3A activity was seen between subjects [70]. However, neither 4βHC nor 6βHCL metabolic ratios were correlated with the oral clearance of midazolam. Although 4βHC and 6βHCL are affected by CYP3A inhibition or induction, they were not reliable predictors of constitutive activity. This study again illustrates the difficulty of using endogenous enzyme activity as a reliable method of assessing oral metabolism and highlights the need for reliable and specific endogenous biomarkers of metabolism and variability. Physiological biomarkers like gastrointestinal microbiome [71] and pH [72] can predict microenvironmental features on drug solubility and stability, and enable pH-sensitive or microbiota-activated release systems to be designed. Recent developments have further demonstrated the role of disease biomarkers in the development of formulation strategies that successfully address gastrointestinal barriers. A triple-emulsion microfluidic core–shell hydrogel system has been designed for the oral delivery of pentoxifylline to treat inflammatory bowel disease (IBD) [73]. The microcapsules, which have a pH response, protected the drug in gastric conditions and targeted the colon for drug delivery, thereby successfully addressing a pH barrier for drug delivery. The treatment significantly reduced the levels of inflammatory biomarkers (IL-1β, IL-6, and TNF-α), improved the integrity of the colon, and corrected beneficial gut microbiota profiles in dextran sulfate sodium (DSS)-induced colitis models. These developments have successfully demonstrated the role of inflammatory and microbiome biomarkers in the design of oral drug delivery systems. Moreover, disease biomarkers provide condition-predicted delivery, i.e., biomarkers of inflammation in inflammatory bowel disease [74] or of biomarker profiles in diabetes [75], to trigger site-specific drug release or dosing adjustment. The use of these biomarkers in the design of drug formulations, apart from enhancing oral bioavailability, abrogates interpatient variability and enhances the accuracy of therapy (Table 1). Biomarker-guided oral delivery systems, therefore, are a promising direction towards personalized medicine, covering the diagnostic knowledge gap with maximized drug therapy.

## 5. Biomarker-Guided Drug Delivery Systems

### 5.1. Concept and Mechanisms

Biomarker-guided drug delivery systems exploit individual biological markers to realize spatial and temporal control over drug release with high specificity, which amplifies the therapeutic response and reduces off-target effects [77]. The safety issues arising due to off-target biological activations are a major problem that needs to be addressed in the context of the translation of biomarker-responsive therapeutic systems. For example, in the context of immune checkpoint inhibitor therapy, unintended biological activations of systemic immune responses have been shown to contribute to immune-related adverse events [78]. These observations underscore the need to establish predictive biomarkers to balance therapeutic and adverse effects. Similarly, unintended biological activations need to be minimized in the context of adaptive drug delivery systems, which should be equipped with established biomarker responses, confirmatory signals, and safety feedback. These are going to be key to the translation of biomarker-responsive therapeutic systems. These systems are engineered to respond to endogenous biomarkers, such as pH change, enzyme activity, metabolite level, or protein expression, which are representative of pathological or physiological conditions at the disease or drug action site. In response to such biomarkers, the delivery platform experiences a physicochemical or biochemical change that initiates drug release [79]. Enzyme-responsive systems take advantage of overexpressed proteases or glycosidases in diseased tissue to break down linker molecules or matrix materials to achieve targeted payload release [80,81]. Metabolite-responsive carriers, for example, release drugs in the presence of glucose in diabetics or lactate in hypoxic tumors. For instance, Fruehauf et al. [82] investigated the design of metabolite-sensitive nanoparticles for targeted therapy. N-isopropylacrylamide (NIPAM) nanoparticles were conjugated to an oxamate derivative to associate lactate dehydrogenase (LDH) to give OxNP–LDH assemblies. These assemblies selectively responded to lactic acid, the signature metabolite of hypoxic tumor microenvironments, by ballooning by up to 65%, but not to related structures. The finding demonstrates a new proof of principle in that nanoparticle sensitivity is adjusted by protein conjugation to allow specific identification of disease-related metabolites and provides a promising approach to increasing bioavailability and therapeutic specificity. Moreover, the incorporation of biomarker recognition elements, such as aptamers [83], antibodies [84], or molecular switches [85], further enhances specificity and responsiveness. Zhu et al. [86] demonstrated a protocol for oral delivery of therapeutic antibodies by use of fluorocarbon-modified chitosan (FCS) as a transmucosal polymeric carrier. Antibodies and FCS together produced nanoparticles that were lyophilized with excipients and enterically coated for oral delivery. FCS facilitated protein transport through transiently rearranging tight junction proteins for systemic absorption. Oral delivery of αPD1 alone or in combination with αCTLA4 at five-fold doses elicited therapeutic outcomes equivalent to those of intravenous injection while sparingly inducing immune-related adverse events. These findings indicate FCS as an exciting platform to revolutionize the oral delivery of protein therapeutics. Mechanistically, these smart systems utilize changes in solubility, swelling, degradation, or conformational structure to control release kinetics in a dynamic manner [87,88]. By combining biomarker sensing and controlled delivery, such sophisticated platforms have great potential to enhance oral bioavailability by targeted release, minimizing systemic exposure, and personalized treatment.

### 5.2. Classes of Biomarker-Responsive Delivery Systems

Biomarker-guided drug delivery systems are a broad class of platforms designed to release therapeutics in response to recognition of distinctive biological signals (Table 2). One well-characterized class is pH-gated systems, which take advantage of pH contrasts in gastrointestinal or pathologically altered tissue pH in order to initiate drug release [89]. In a previous study [89], Sun et al. demonstrated that worm-like core–shell hybrid nanoparticles comprising mesoporous silica cores and polymer shells loaded with 3-acrylamidophenylboronic acid (AAPBA) and NIPAM as sensor pendant groups can be efficiently loaded up to 15% with temperature-responsive swelling/collapse behavior. In vitro results demonstrate glucose-dependent release of insulin that is pH-tunable with cross-linked shells (Dex-Ma), giving systematically higher release rates for longer times in comparison to non-cross-linked shells. Cell viability tests substantiated favorable biocompatibility. The results demonstrate the promise of pH-gated, glucose-sensitive nanoparticles as an exciting delivery vehicle for self-adjusting insulin delivery. Polymers with embedded acid-cleavable linkages or ionizable groups, for instance, can swell or hydrolyze selectively in acidic microenvironments like tumors or inflamed gut patches, to provide targeted therapy [90]. The second class includes enzyme-responsive platforms that are intended to be responsive to elevated concentrations of enzymes such as proteases, phospholipases, or glycosidases in disease states [91]. These systems incorporate enzyme-cleavable substrates or linkers within their matrix so that the drug gets delivered in a site-specific manner [92]. Metabolite-sensitive platforms are responsive to metabolites such as glucose or lactate so that dynamic drug delivery is achieved for metabolic disease or hypoxic tumors. For example, glucose-sensitive hydrogels regulate insulin release in diabetic patients [76]. For oral delivery of insulin, Ying et al. [76] reported a dual-responsive hydrogel system that can enhance bioavailability and mimic endogenous regulation. The hydrogel formulated by chemically modifying carboxymethyl agarose via 3-amino-phenylboronic acid and L-valine (CPL) demonstrated excellent biocompatibility and competent insulin encapsulation. Insulin-loaded hydrogel (Ins-CPL) demonstrated controlled release of insulin in the presence of physiological glucose and pH changes to facilitate higher utilization and intestinal uptake. Ins-CPL demonstrated the capability of effectively controlling blood glucose for longer durations in diabetic rats by overcoming the intestinal barrier and by actively responding to metabolic stimuli. These results indicate that CPL hydrogels hold promise for oral insulin therapy. In addition, protein/antibody-responsive systems make use of molecular recognition elements, including aptamers or antibodies, to recognize disease-linked biomarkers, inducing conformational change to release the drug cargo [93]. Hybrid systems of novel design merge a variety of triggers to improve selectivity and regulation [94]. These systems have complex mechanisms, e.g., swelling, degradation, or molecular switching, to create targeted and adaptive drug delivery with greatly improved therapeutic action, particularly for oral delivery where heterogeneity of biomarkers prevails.

### 5.3. Engineering Approaches

Biomarker-directed oral drug delivery system design combines materials science, nanotechnology, and molecular engineering to achieve targeted, responsive drug release. The most universally used nanocarriers are polymeric nanoparticles, liposomes, micelles, and solid lipid nanoparticles because of their tunable physicochemical properties and versatility in drug entrapment [95]. Biomarker-recognizing moieties, including aptamers, antibodies, or peptides, attached to the carriers facilitate selective targeting and stimulus-induced release upon biomarker binding [96,97,98]. Stimuli-responsive polymers are another significant engineering technique, wherein cleavable or modulated side linkers or chain linkers are achieved by enzymatic response, pH change, or metabolite level [99]. These polymers degrade, swell, or change conformation when contacted with the biomarker, allowing drug liberation to be released in the GI tract [100]. Additionally, molecular switches and self-immolative linkers enable cascade release mechanisms, where biomarker recognition initiates a chain reaction leading to payload release [101,102]. Delivery products for oral administration also utilize mucoadhesive or mucus-penetrating properties to enhance residence time and contact at target sites [103]. Incorporation into wearables and biosensors also provides for real-time monitoring and dose adjustment [104]. Emerging studies provide early proof-of-principle for real-time biomarker-informed dose optimization. In a stress-induced diabetic model using continuous subcutaneous microdialysis, rapid changes in pH, lactate, and glucose were observed under physiological stress, and long-acting insulin shortened stress-related metabolic perturbations, supporting multi-biomarker-guided adaptive therapy [105]. Importantly, human validation has been demonstrated using a wearable microneedle-based continuous biomarker/drug monitoring (MCBM) system capable of simultaneous real-time measurement of glucose and metformin in interstitial fluid [106]. This platform enables integrated pharmacokinetic–pharmacodynamic assessment and has shown safety and precision suitable for therapeutic adjustment. Together, these findings support the translational feasibility of biomarker-guided adaptive dosing in personalized medicine. Collectively, these engineering techniques provide a refined strategy for addressing oral bioavailability challenges through biomarker-responsive, patient-adaptive drug delivery systems.

## 6. Strategies for Oral Bioavailability Enhancement in Biomarker-Guided Systems

Oral bioavailability enhancement via biomarker-guided drug delivery involves the combination of classical formulation approaches with biomarker-responsive triggers to overcome physical and biological barriers [107]. Solubility and dissolution are enhanced through the application of amorphous solid dispersions [108], lipid formulations [109], and nanocrystals [110] engineered to release the drug in response to specific biomarkers, such as pH or enzymatic activity in the gut lumen. A recent phase 1 open-label trial by Serebrenik et al. [111] investigated an amorphous solid dispersion of genistein produced by hot melt extrusion (genistein HME) in 34 healthy volunteers. In both the single ascending dose (500–3000 mg) and multiple daily dose (3000 mg/day for six days) studies, genistein HME was well tolerated, with no dose-limiting toxicities and only mild-to-moderate gastrointestinal events. Pharmacokinetic profiling revealed a marked increase in bioavailability between 2000 mg and 3000 mg, while the no observable adverse effect level was 500 mg. Importantly, gene expression biomarkers identified through RNA sequencing demonstrated drug-related transcriptional changes 8–12 h after repeated dosing, providing a pharmacodynamic signature of systemic activity. Based on safety, exposure, and biomarker responses, the putative effective human dose was established at 3000 mg.

In another aspect, permeability increases via the application of permeation enhancers or transporter-targeting ligands guided by biomarker profiles indicative of transporter function or expression [112]. Such site-specific modulation raises transcellular or paracellular transport with a simultaneous decrease in systemic toxicity. Avoidance of drug degradation via enzymes and first-pass metabolism is overcome via co-delivery of inhibitors of enzymes or prodrug designs that are activated in response to metabolic biomarkers. Additionally, site-specific and controlled release formulations utilize biomarkers such as localized pH gradients or disease-specific enzymes to release the drug precisely where absorption is maximal, subsequent to a reduction in systemic exposure and toxicity [113]. Muco-adhesion [114] and mucus penetration [115] also enhance intestinal residence time and foster proximity to the absorptive epithelium. Collectively, such approaches in combination with real-time monitoring of biomarkers provide a patient-specific strategy for optimizing oral drug absorption, decreasing variability, and optimizing therapeutic effect.

## 7. Current Applications and Case Studies

Biomarker-regulated oral drug delivery systems have been promising across multiple therapeutic applications and have the potential to transform personalized medicine. A classic example is glucose-responsive insulin release systems in diabetes, where biomarker sensing can provide demand-release insulin, closely simulating physiological regulation and thereby enhancing glycemic control [116,117]. Research by Yu et al. [118] presented an oral insulin system that is glucose-sensitive for controlling postprandial blood glucose. The system is made up of Fc receptor (FcRn)-targeted liposomes coated with a glucose-responsive hyaluronic acid (HA) shell on the surface. Elevated intestinal glucose induces the removal of phenylboronic acid-modified HA, revealing Fc groups that enhance intestinal uptake through FcRn-mediated transport. The system regulated postprandial glucose in type 1 diabetic mice effectively through targeted, demand-modeled insulin release. Of particular importance, it is the first oral insulin delivery system that is activated by postprandial glucose biomarkers, with the potential to be used as a simple diabetes control regimen.

In cancer, pH- and enzyme-sensitive oral formulations target the acidic and protease-rich tumor microenvironment, providing site-specific delivery of chemotherapy with lower systemic toxicity [119]. Recently, Palmer et al. [120] investigated targeted therapies based on biomarkers to replace standard chemotherapy for advanced ovarian cancer. At the patient-derived xenograft (PDX) level, modeling human tumors, 21 mono- and combination therapies were tested. Three monotherapies and one combination exhibited activity for defined PDX subsets. Gene expression biomarkers afterwards predicted responsiveness to therapy by these agents, despite none being directed at oncogenic drivers per se. While agents individually proved less efficient compared to chemotherapy, close to 90% PDXs, including those chemotherapeutically resistant, proved responsive to at least one biomarker-guided therapy. The Cancer Genome Atlas (TCGA) data validation agreed with biomarker occurrence at the patient level, supporting precision-based therapy for ovarian cancer.

In inflammatory bowel disease, biomarker-responsive delivery systems, such as reactive oxygen species (ROS) or cytokine-responsive systems, allow localized delivery of medication to achieve enhanced efficacy while minimizing adverse effects. Kumari et al. [121] created a ROS-activatable nanogel platform for site-specific ulcerative colitis treatment, releasing polymeric chloroquine (PCQ), a hydroxychloroquine analog. Nanogels (180–680 nm; +13 to +24 mV) were degradable in ROS-abundant environments, stable in gastrointestinal fluids, and selectively targeted colonic inflamed tissue. In a colitis mouse model, nanogels also surpassed hydroxychloroquine (HCQ) in inducing histological cure, epithelial healing, immune infiltration suppression, and STAT3 activation suppression, an inflammatory hallmark biomarker. Cytokine/eicosanoid profiling elicited robust local and incomplete systemic immunomodulation, and T1 exhibited best-in-class local activity. The findings position biomarker-responsive nanogels as a very promising, safer therapeutic intervention for ulcerative colitis.

**Figure 2:**
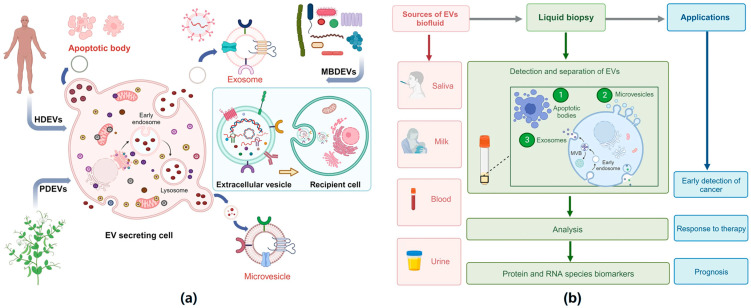
Extracellular vesicles (EVs) as biomarkers and therapeutic carriers in biomarker-guided drug delivery. (**a**) Biogenesis and sources of EVs. Cells from diverse origins, including human tissues, plants, and microorganisms, release apoptotic bodies, microvesicles, and exosomes through distinct intracellular pathways. EVs encapsulate proteins, nucleic acids, and lipids that mirror the molecular signatures of parent cells, enabling intercellular communication and providing inherent biomarker cargo for targeted drug delivery applications. (**b**) EVs in liquid biopsy and clinical applications. EVs are abundantly present in body fluids such as saliva, milk, blood, and urine, allowing non-invasive isolation via liquid biopsy. Molecular analysis of EV-associated biomarkers supports early disease detection, monitoring of therapeutic response, and prognosis assessment, positioning EVs as powerful platforms for biomarker-guided precision medicine and targeted drug delivery. Adapted from [122] under the Creative Commons license CC BY 4.0 (http://creativecommons.org/licenses/by/4.0/, accessed on 28 January 2026).

Transporter biomarker-based strategies have been applied in the case of optimizing orally administered anticancer and antiviral medication by modulating efflux transporter function or taking advantage of uptake transporters [122]. Kumar et al. emphasized the function of extracellular vesicles (EVs) as key intercellular mediators of communication and possible biomarkers for disease. EVs transfer proteins, nucleic acids, and lipids and impact many pathological processes, such as cancer development and drug resistance (Figure 2). Another notable observation by Ma et al. [123] is that adriamycin-resistant breast cancer cells secrete TrpC5-carrying EVs that trigger P-glycoprotein expression, leading to drug resistance in target cells. Silencing TrpC5 suppressed EV secretion, whereas TrpC5-positive EVs were highly expressed in human breast cancer tissues and mouse models. These findings indicate that TrpC5-positive EVs are predictive biomarkers for chemotherapy response and targets for overcoming drug resistance.

Co-delivery of enzymatic inhibitors with medication by metabolic biomarker signature has also optimized bioavailability in cases of high first-pass clearance [124,125]. Kennedy et al. [126] investigated the utility of Aβ40, Aβ42, and sAPPβ as pharmacodynamic biomarkers of beta-site amyloid precursor protein cleaving enzyme 1 (BACE1) inhibition in Alzheimer’s disease (AD). Verubecestat (MK-8931), an extremely potent and selective BACE1 inhibitor, decreased these biomarkers in plasma, cerebrospinal fluid (CSF), and brain by substantial amounts after acute and chronic dosing in rats, monkeys, and humans. Most importantly, chronic dosing was attainable at multiples many times greater than during clinical assessment with no serious adverse effects, justifying the biomarker-based safety window. Reductions in CSF biomarkers in healthy volunteers and AD patients validated target engagement, and pathway modeling informed Aβ pool dynamics. The results underscore the utility of biomarker-guided approaches to optimize dose selection and hasten clinical advance.

Many of these strategies have advanced to preclinical and early clinical phases and are proven to improve pharmacokinetic profiles and show efficacy. These case histories illustrate the revolutionary promise of biomarker-guided delivery to orally deliver therapy in a patient-bio-matched manner and avenues to expanded clinical translation and next-generation orally administered medication.

## 8. Regulatory and Translational Considerations

Bench-to-bedside translation of biomarker-based oral drug delivery devices needs to consider clinical validation and regulatory pathways. Drug regulatory agencies like the U.S. Food and Drug Administration (US FDA) and European Medicines Agency (EMA) stress stringent qualification and validation of biomarkers used for patient stratification, monitoring therapy, and triggering device activation for safety, efficacy, and reproducibility [127,128,129]. Standardization of biomarker assays and a clear correlation of biomarker levels with clinical endpoints are critical for receiving regulatory clearance [130]. Manufacturing challenges arise owing to the complexity of incorporating biomarker-responsive materials, such as the need for scalable and repeatable processes compliant with Good Manufacturing Practices (GMP) [131,132]. Also, companion diagnostics might need to be co-developed alongside biomarker-driven therapeutics, involving traversal of additional regulatory routes [133]. Ethical considerations related to patient selection, privacy, and informed consent must be addressed, especially when biomarker data influences treatment decisions [134]. Despite these obstacles, new guidance notes from regulators acknowledge the promise of biomarker-guided precision medicines and outline routes for expedited approval. Dialog among formulation scientists, clinicians, regulatory experts, and industry partners must then play a key role in overcoming these challenges. Proactively engaging regulations and aspects of translation in early stages of product development will, however, accelerate the use of biomarker-guided oral delivery devices in the clinic, ultimately allowing for tailored and efficient therapeutic interventions.

## 9. Challenges, Knowledge Gaps, and Future Perspectives

In spite of remarkable progress, several limitations and knowledge gaps impede the high-speed implementation of biomarker-guided oral drug delivery systems. Major roadblocks are biomarker identification and qualification as clinically significant biomarkers with reproducible capacity to anticipate drug absorption, metabolism, and outcome of therapy in variable populations [135]. Interpatient variability and the dynamic nature of biomarker expression hinder the effective design of drug delivery systems. Biomarker variability and patient heterogeneity are still important issues to overcome for adaptive therapeutic systems. A quantitative approach to manage these issues has been proposed using quantitative systems pharmacology (QSP) modeling, which combines different scales of biological information to simulate the dynamics of the tumor and immune system, as well as the effects of the drugs [136,137]. QSP modeling has been proposed for the optimization of immunotherapy, the prediction of the first-in-human dose, the identification of responders, and the minimization of side effects such as cytokine release syndrome. QSP modeling can quantitatively account for the heterogeneity of the immune response between patients, thereby improving the adaptive therapeutic approach. Furthermore, the intricacy of translating biomarker-responsive materials into low-cost, large-scale oral forms continues to be a leading manufacturing obstacle [138]. Scalability of manufacturing is another key issue to be addressed for translational research of biomarker-based and adaptive drug delivery systems. For example, scaling up the production of intelligent materials and fully integrated platforms containing sensors and actuators should ensure reproducibility and batch-to-batch consistency. This issue is further complicated for multifunctional platforms containing sensing and processing capabilities. For hybrid drug delivery platforms containing devices and drugs, the simultaneous pharmacological and pharmacotechnical validation of the drug and the device can represent a limitation to the translational process under current Good Manufacturing Practice (cGMP) regulations. Inadequate knowledge of the long-term biocompatibility and safety of new biomaterials and nanocarriers also constitutes a translational threat [139]. Next-generation biosensing systems, including wearable devices, must be the focus of future research to realize real-time, dynamic monitoring and feedback-controlled drug delivery through the development of multiplexed biomarker panels [140,141]. Application of machine learning and artificial intelligence (AI) to model big biomarker data sets provides hopeful prospects in dose optimization and tailor-made formulation design [142]. Recent developments have shown the feasibility of the practical integration of AI and wearable biomarker monitoring systems. For instance, a study by Cao et al. [143] utilized machine learning models to analyze the continuous glucose monitoring system for the precise prediction of glucose level changes among patients with septicemia. The PatchTST and DLinear models have been found to be precise in predicting glucose changes with minimal prediction errors. The glucose levels among the patients with septicemia act as a biomarker, which can be constantly monitored and analyzed with the aid of AI for the prediction of changes in the biomarker. Additionally, advancing synthetic biology and molecular engineering can provide next-generation “smart” delivery systems with greater sensitivity and specificity [144]. Spanning interdisciplinary expertise and regulatory cooperation will be paramount in overcoming current hurdles. Regulatory harmonization is another major challenge. In the case of adaptive and semi-autonomous dose-adjusting systems, there is a lack of clear precedential guidelines, especially when such systems are classified as combination products involving drugs, devices, and software. In this context, the need for clear validation processes, including the assessment of performance consistency across a wide range of patient groups, is a major requirement. In addition, the need for a global evaluation and approval pathway, as well as a system of post-marketing surveillance, is a key requirement to ensure that such systems are regulated effectively. Finally, the intersection of biomarker science, sophisticated delivery technologies, and digital health capabilities can transform oral therapeutics into highly personalized, adaptive, and efficacious treatment regimens. It should be noted that the majority of biomarker-responsive oral delivery systems remain at the preclinical stage, underscoring the need for rigorous clinical validation and biomarker qualification to enable translation.

## 10. Conclusions

Biomarker-driven delivery of drugs is the next frontier of the journey towards precision oral medicine. Through leveraging the interplay of physiological and molecular indices and novel formulation approaches, these delivery systems surmount highly dynamic oral bioavailability barriers such as poor solubility, permeability barriers, enzymatic degradation, and first-pass clearance. Dynamic regulation of drug delivery according to personalized biomarkers has tremendous promise for minimizing inter-subject variability, optimizing therapeutic outcomes, and limiting unwanted side effects. Newly developed biomarker-sensitive materials, nanocarriers, and biosensing platforms have shown promising preclinical and clinical efficacy across a range of diseases, from diabetes, oncology, to inflammatory disease. However, success in clinical translation necessitates overcoming challenges of biomarker validation, manufacturing scalability, regulatory approval, and long-term safety guarantees. Future integration of AI, multiplex biosensors, and intelligent delivery platforms has the promise to facilitate the rapid transition of next-generation oral therapeutics to be highly context-responsive and highly personalized. With the advancement in biomarker science and delivery technology of drugs on an ongoing basis, biomarker-driven oral delivery of drugs has the promise to transform therapeutic interventions with more efficient and patient-specific ones.

## Figures and Tables

**Table 1:** Mechanism-Driven Biomarker-Responsive Oral Drug Delivery Platforms and Their Translational Progress.

Biomarker Type	Representative Biomarker	Associated Barrier	Delivery Strategy	Experimental Model	Key Findings	Translational Status	Ref.
Transporter Biomarker	OAT1/OAT1/3 (Taurine, Pyridoxic acid)	Renal secretion variability	Transporter-guided dosing optimization	Rat in vivo PK	Calcitriol alters OAT-mediated disposition; taurine sensitive indicator	Translational preclinical	[63]
Transporter Biomarker	OCTN1 (Ergothioneine)	Tissue distribution barrier	Transporter-aware exposure adjustment	Rat in vivo PK	Altered AUC, clearance, and tissue partitioning	Translational preclinical	[64]
Transporter Biomarker	OCTN2 (L-carnitine)	Tissue distribution barrier	Transporter-aware exposure adjustment	Rat in vivo PK	Altered AUC, clearance, and tissue partitioning	Translational preclinical	[65]
Transporter Biomarker	OCT2 (Creatinine)	Renal elimination variability	DDI risk prediction	Rat in vivo PK	Reduced renal clearance following modulation	Clinically relevant endogenous marker	[65]
Transporter Biomarker	OATP1B1 (Coproporphyrin I)	Hepatic uptake limitation; DDI risk	Dose adjustment; transporter-informed formulation selection	Clinical study	CP-I correlates with OATP1B1 inhibition and DDI magnitude	Clinically validated endogenous biomarker	[67]
Metabolic Enzyme Biomarker	CYP3A (4β-hydroxycholesterol)	First-pass metabolism	Enzyme-guided prodrug design; inhibitor co-administration	Human phenotyping study	Sensitive to induction/inhibition but limited for basal activity prediction	Clinically used for induction monitoring	[70]
Metabolic Enzyme Biomarker	UGT isoforms	Phase II metabolism variability	Metabolism-resistant formulations	3D renal cell model	Enhanced expression in physiologically relevant model	Preclinical mechanistic	[50]
Inflammatory Biomarker	IL-6, TNF-α, IL-1β	Inflamed intestinal microenvironment	pH-responsive hydrogel microcapsules	DSS-induced colitis model (mice)	Reduced inflammatory markers; improved mucosal integrity	Preclinical proof-of-concept	[73]
Microbiome Biomarker	Gut dysbiosis profile (e.g., *Bacteroides* spp.)	Microbiota-mediated metabolism	Microbiota-responsive release systems	IBD mouse model	Restored microbial balance; targeted colonic delivery	Emerging preclinical	[73]
Metabolite Biomarker	Glucose	Hyperglycemic microenvironment	Glucose-responsive hydrogels	Diabetic rat model	Controlled insulin release; prolonged glucose control	Advanced preclinical	[76]
pH Biomarker	Colonic pH (≈7.5)	Gastric degradation	Core–shell pH-responsive capsules	In vivo colitis model	Gastric protection; colonic-specific release	Preclinical translational	[73]

**Table 2:** Classes of Biomarker-Responsive Drug Delivery Systems.

Class of System	Trigger/Biomarker	Mechanism of Drug Release	Representative Example	Key Outcomes/Advantages	Ref.
pH-responsive systems	pH changes in GI tract, tumors, inflamed tissues	Swelling or hydrolysis of polymers under acidic conditions	Core–shell nanoparticles with mesoporous silica and polymer shells (AAPBA/NIPAM)	pH-tunable, glucose-dependent insulin release; enhanced biocompatibility; promising for self-regulated insulin delivery	[89,90]
Enzyme-responsive systems	Disease-associated enzymes (proteases, phospholipases, glycosidases)	Enzyme-cleavable linkers or substrates degrade matrix	Enzyme-sensitive polymeric carriers	Site-specific drug release in enzyme-rich disease microenvironments	[91,92]
Metabolite-responsive systems	Metabolites (e.g., glucose, lactate)	Metabolite-induced swelling or structural changes	Glucose-sensitive CPL hydrogel for oral insulin delivery	Controlled insulin release, improved bioavailability, prolonged glucose regulation in diabetic models	[76]
Protein/antibody-responsive systems	Disease-related proteins or biomarkers	Molecular recognition via antibodies or aptamers induces conformational changes	Aptamer- or antibody-functionalized carriers	High specificity toward disease biomarkers; triggered cargo release	[93]
Hybrid multi-responsive systems	Combination of pH, enzymes, metabolites, or proteins	Integrated mechanisms (swelling, degradation, molecular switching)	Multi-stimuli responsive nanocarriers	Enhanced selectivity, adaptive release, improved therapeutic efficacy—especially for oral delivery	[94]

AAPBA: 3-acrylamidophenylboronic acid, NIPAM: N-isopropylacrylamide, CPL: Carboxymethyl agarose modified with 3-amino-Phenylboronic acid and L-valine.

## Data Availability

No new data were created or analyzed in this study.

## Abbreviations

The following abbreviations are used in this manuscript:

AAPBA	3-acrylamidophenylboronic acid
AD	Alzheimer’s disease
AI	artificial intelligence
BACE1	beta-site amyloid precursor protein cleaving enzyme 1
BCS	Bio-pharmaceutics Classification System
CPL	Carboxymethyl agarose modified with 3-amino-Phenylboronic acid and L-valine
CSF	cerebrospinal fluid
ELISA	enzyme-linked immunosorbent assays
EMA	European Medicines Agency
EVs	extracellular vesicles
FCS	fluorocarbon-modified chitosan
GI	gastrointestinal
GMP	Good Manufacturing Practices
HA	hyaluronic acid
HCQ	hydroxychloroquine
HME	hot melt extrusion
Ins-CPL	Insulin-loaded hydrogel
LC-MS/MS	liquid chromatography–tandem mass spectrometry
LDH	lactate dehydrogenase
MRI	magnetic resonance imaging
NGS	next-generation sequencing
NIPAM	N-isopropylacrylamide
OATP	organic anion transporting polypeptides
PCQ	polymeric chloroquine
PCR	polymerase chain reaction
PDX	patient-derived xenograft
PET	positron emission tomography
ROS	reactive oxygen species
TCGA	The Cancer Genome Atlas
UGTs	UDP-glucuronosyltransferases
US FDA	U.S. Food and Drug Administration
